# Human γS-Crystallin Mutation F10_Y11delinsLN in the First Greek Key Pair Destabilizes and Impairs Tight Packing Causing Cortical Lamellar Cataract

**DOI:** 10.3390/ijms241814332

**Published:** 2023-09-20

**Authors:** Venkata Pulla Rao Vendra, Christian Ostrowski, Marzena A. Dyba, Sergey G. Tarasov, J. Fielding Hejtmancik

**Affiliations:** 1 Ophthalmic Molecular Genetics Section, Ophthalmic Genetics and Visual Function Branch, National Eye Institute, National Institutes of Health, Bethesda, MD 20892, USA; pullaraovv@gmail.com (V.P.R.V.);; 2 Biophysics Resource in the Center for Structural Biology, National Cancer Institute, National Institutes of Health, Frederick, MD 21702, USA; marzena.dyba@nih.gov (M.A.D.); tarasovs@mail.nih.gov (S.G.T.)

**Keywords:** cataract, crystallins, aggregation, Greek key motif, F10_Y11delinsLN, genotype-phenotype correlation

## Abstract

Aromatic residues forming tyrosine corners within Greek key motifs are critical for the folding, stability, and order of βγ-crystallins and thus lens transparency. To delineate how a double amino acid substitution in an N-terminal-domain tyrosine corner of the CRYGS mutant p.F10_Y11delinsLN causes juvenile autosomal dominant cortical lamellar cataracts, human γS-crystallin c-DNA was cloned into pET-20b (+) and a p.F10_Y11delinsLN mutant was generated via site-directed mutagenesis, overexpressed, and purified using ion-exchange and size-exclusion chromatography. Structure, stability, and aggregation properties in solution under thermal and chemical stress were determined using spectrofluorimetry and circular dichroism. In benign conditions, the p.F10_Y11delinsLN mutation does not affect the protein backbone but alters its tryptophan microenvironment slightly. The mutant is less stable to thermal and GuHCl-induced stress, undergoing a two-state transition with a midpoint of 60.4 °C (wild type 73.1 °C) under thermal stress and exhibiting a three-state transition with midpoints of 1.25 and 2.59 M GuHCl (wild type: two-state transition with C_m_ = 2.72 M GuHCl). The mutant self-aggregates upon heating at 60 °C, which is inhibited by α-crystallin and reducing agents. Thus, the F10_Y11delinsLN mutation in human γS-crystallin impairs the protein’s tryptophan microenvironment, weakening its stability under thermal and chemical stress, resulting in self-aggregation, lens opacification, and cataract.

## 1. Introduction

To transmit and focus light onto the retina, the human eye lens needs to be transparent with a high refractive index of approximately 1.33 [1]. To obtain these two key features, the microarchitecture of the lens is avascular, with a regular array of elongated fiber cells devoid of organelles and filled with high concentrations of highly stable proteins termed lens crystallins. Crystallins make up over 90% of the total lens protein content and are thought to maintain the favorable optical conditions of the lens with their compact globular structures, short-range order, and packing. Together, these result in a constant refractive index over distances approximating the wavelength of the transmitted light and hence transparency [2,3].

Ubiquitous crystallins include two large gene families, the α and βγ-crystallins. α-crystallins are members of the small heat shock protein family and act as chaperones [4], while βγ-crystallins are structural proteins related to spore coat proteins [5]. βγ-crystallins comprise two domains, each domain consisting of two Greek key motifs, and a super-secondary structural fold. Each βγ-crystallin Greek key motif consists of four β-sheets which run antiparallel to each other and form a 3,1 type of Greek key motif [6]. Human γS-crystallin is a 178-amino-acid protein with a total of 27 aromatic residues (fourteen tyrosines Y, four tryptophans W, and nine phenylalanines F). These aromatic residues play an important role in stabilizing the Greek key fold by participating in “Greek key” and “non-Greek key” pairs or by forming “Tyrosine corners” [7].

Five aromatic pairs are present in β-hairpin sequences in human γS-crystallin. Of these, four pairs exist in Greek key motifs and are termed Greek key pairs (Y11-F16, Y50-F55, F99-F104, and Y140-Y145; the initial methionine is counted) and one falls outside the Greek key motifs, termed a non-Greek key pair (Y21-Y33). Three of the homologous aromatic pairs (two Tyr/Tyr, one Phe/Phe) and two of the non-homologous aromatic pairs (Tyr/Phe) are present in the β-hairpins of the human γS-crystallin protein. Energetically, the optimal distance between these aromatic rings is 4.5–7 Å, either perpendicular or parallel to each other [8]. They contribute a free energy of 0.6–1.3 kcal mol^−1^ to the protein when they are perpendicular to each other [8] or about 0.75 kcal mol^−1^ stability to the protein by parallel displacement stacking [9].

Any mutation that substitutes a non-aromatic residue for the crucial phenylalanine or tyrosine in an aromatic pair is predicted to destabilize βγ-crystallin folding severely. A dinucleotide substitution NM_017541.4:c.30_31delinsAA resulting in a NP_060011.1:p.(F10_Y11delinsLN) substitution in human γS-crystallin has been reported to be associated with autosomal dominant cortical lamellar cataracts with juvenile onset [10]. This two-amino-acid substitution disrupts the interaction of tyrosine and phenylalanine in the first Greek key pair (Y11-F16), and in addition, the adjacent F10 is replaced by a hydrophobic leucine residue. In this study, we investigated the role of the F10_Y11delinsLN change in the destabilization of γS-crystallin and subsequent cataract formation. This change impairs the protein’s tryptophan microenvironment and increases its surface hydrophobicity under physiological conditions and decreases its stability, leading to early denaturation under both thermal and chemical stress. This eventually results in the formation of high-molecular-weight (HMW) aggregates, causing lens opacification after overcoming the main defense systems of the lens against cataract [10] including the strong reducing environment sustained by high levels of glutathione [11] and the chaperone activity of αA-crystallin [12].

## 2. Results

### 2.1. Backbone Conformation of Wild-Type and F10_Y11delinsLN Mutant γS-Crystallin

**Figure 1:**
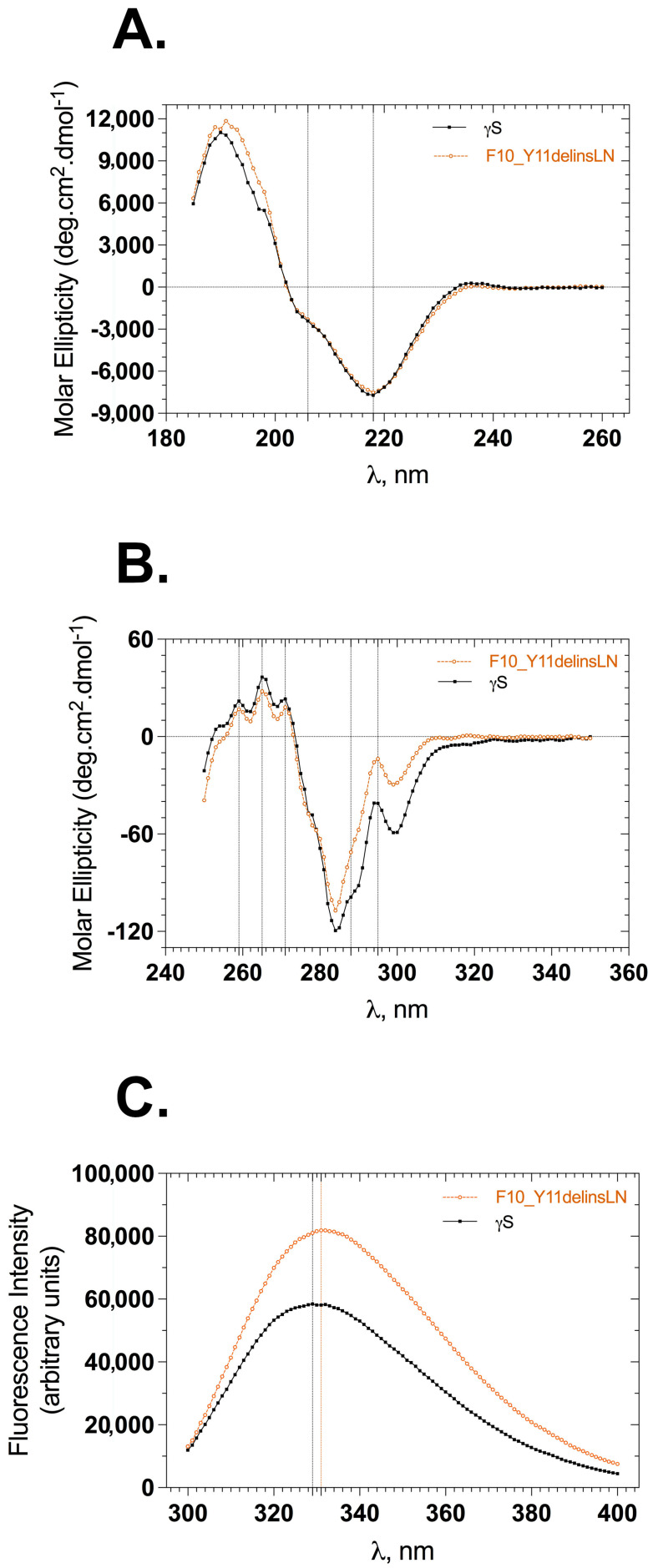
Secondary and tertiary structural features of wild-type (black squares) and F10_Y11delinsLN mutant (brown circles) human γS-crystallin. (**A**) Far-UV CD spectra. Dotted vertical lines at 218 nm and 206 nm represent the characteristic wavelengths of β-sheets and α-helices, respectively. (**B**) Near-UV CD spectra. Dotted vertical lines represent tryptophan (295 nm), tyrosine (288 nm), and phenyl alanine (271, 265, 259 nm) CD maxima in the proteins. (**C**) Tryptophan emission fluorescence spectra. λ_exc_: 295 nm; dotted vertical lines represent emission maxima of the proteins.

The backbone conformation of γS-crystallin samples was monitored by following the circular dichroism (CD) in the far-UV region (185–260 nm). The far-UV CD spectrum of γS-crystallin displays a strong negative band at 218 nm and a small arm around 206 nm, both of these peaks being characteristic indicatives of antiparallel β-sheet and α-helices (Figure 1A). Wild-type and mutant spectra are almost identical, overlaid well, and show slightly varying molar ellipticities at characteristic peaks. The mutant shows a molar ellipticity of −7510 units at 218 nm and −2297 units at 206 nm, while the wild type shows −7716 and −2409 units at 218 and 206 nm, respectively. This is not a significant change and indicates that the overall backbone conformation of the mutant does not vary from that of the wild-type protein. To check the stability of wild-type and mutant proteins under GuHCl-induced stress, both proteins were incubated with varying concentrations of GuHCl (0–5 M) for 12–14 h, and far-UV CD spectra were recorded. The wild-type β-sheet conformation was relatively stable through 2 M GuHCl (Figure S1A), while the mutant displayed a somewhat stable β-sheet conformation only through 1 M GuHCl (Figure S1B); beyond this point the mutant’s backbone conformation is mostly lost. We were unable to record CD spectra below 206 nm with GuHCl samples due to high-tension voltages.

### 2.2. Tryptophan Microenvironments of Wild-Type and F10_Y11delinsLN Mutant γS-Crystallin

Details of the tertiary structure were assessed by measuring the circular dichroism of each protein in the near-UV range (250–350 nm) and recording the tryptophan fluorescence in the 300–400 nm range by selectively exciting the protein at 295 nm. Wild-type and mutant near-UV CD spectra showed similar peaks with differing intensities of molar ellipticities (Figure 1B). This difference was greatest between 285 and 310 nm: −41.0 units for the wild type and −13.8 units for the mutant at 295 nm, and −98.8 units and −71.2 units at 288 nm for the wild-type and mutant, respectively. Differences in the molar intensities at the 271, 265, and 259 nm peaks were less pronounced than those at the 288 and 295 nm peaks, consistent with an altered microenvironment around tryptophan and tyrosine residues in the protein. The tertiary structure was also assessed using tryptophan fluorescence emission spectra. Human γS-crystallin has four buried tryptophans at positions 47, 73, 137, and 163 (the first methionine is counted), whose fluorescence is efficiently quenched in the native state compared to the denatured stated [13]. The F10_Y11delinsLN mutant shows approximately 1.4 times greater tryptophan fluorescence with a 2 nm redshift (from 329 to 331 nm) compared to the wild type (Figure 1C). This further confirms that the microenvironment around the tryptophans is slightly altered in the mutant. The tertiary structure under GuHCl-induced stress was recorded to assess the stability of the mutant compared to the wild-type protein. Both proteins emitted less fluorescence at 1 M GuHCl compared to their native fluorescence (Figure S2A,B). The emission maxima of the wild type were 332, 332, and 336 nm at 0, 1, and 2 M GuHCl, suggesting that, in the wild-type protein, the tryptophan microenvironments are intact through 2 M GuHCl, whereas the mutant showed emission maxima of 334, 338, and 349 nm at 0, 1, and 2 M GuHCl, respectively, further indicating alteration of the mutants’ tryptophan microenvironment by 1 M GuHCl. Both proteins were completely unfolded by 3 M GuHCl and showed increased tryptophan fluorescence relative to their native forms.

### 2.3. Surface Hydrophobicity of Wild-Type and F10_Y11delinsLN Mutant γS-Crystallin

**Figure 2:**
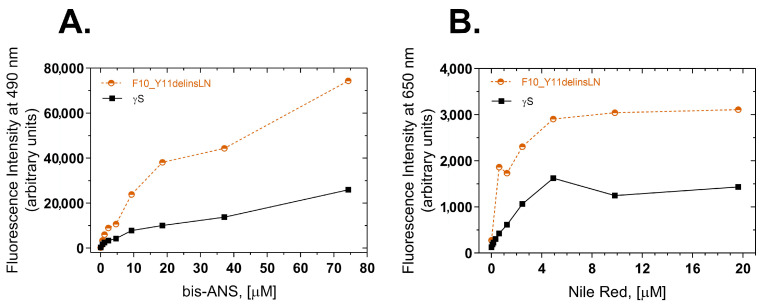
Surface hydrophobicity of wild-type (black squares) and F10_Y11delinsLN mutant (brown circles) human γS-crystallin estimated by bis-ANS and Nile Red fluorescence. (**A**) bis-ANS emission spectra. λ_exc_: 390 nm; λ_emission_ value at 490 nm was used for the plots. (**B**) Nile Red emission spectra. λ_exc_: 540 nm; λ_emission_ value at 650 nm was used to plot the graphs.

To further investigate the exposure of hydrophobic amino acids to the protein surface, two extrinsic probes, bis-ANS (4,4′-Dianilino-1,1′-binaphthyl-5,5′-disulfonic acid) [14] and Nile Red (9-diethylamino-5-benzo phenoxazine) [15], were used to measure the surface hydrophobicity of the wild-type and F10_Y11delinsLN mutant proteins. bis-ANS Binds to hydrophobic patches on the surface of proteins and the resulting protein dye complex emits a fluorescence at 490 nm upon excitation at 390 nm. The F10_Y11delinsLN mutant shows approximately a three-fold greater fluorescence across a broad range of bis-ANS concentrations (Figure 2A), indicating that the mutant has more surface hydrophobicity than the wild-type protein. Nile Red, another hydrophobic dye, also binds to exposed hydrophobic regions of proteins in a similar manner, and, when bound, emits a fluorescence with a maximum at 650 nm after being excited at 540 nm. The F10_Y11delinsLN mutant shows approximately 2.4 times greater fluorescence with Nile Red across a wide range of dye concentrations (Figure 2B), further confirming the increase in hydrophobic patches on the F10_Y11delinsLN mutant’s surface relative to wild-type γS-crystallin, even under benign conditions.

### 2.4. Stability of Wild-Type and F10_Y11delinsLN Mutant γS-Crystallin under Thermal Stress

**Figure 3:**
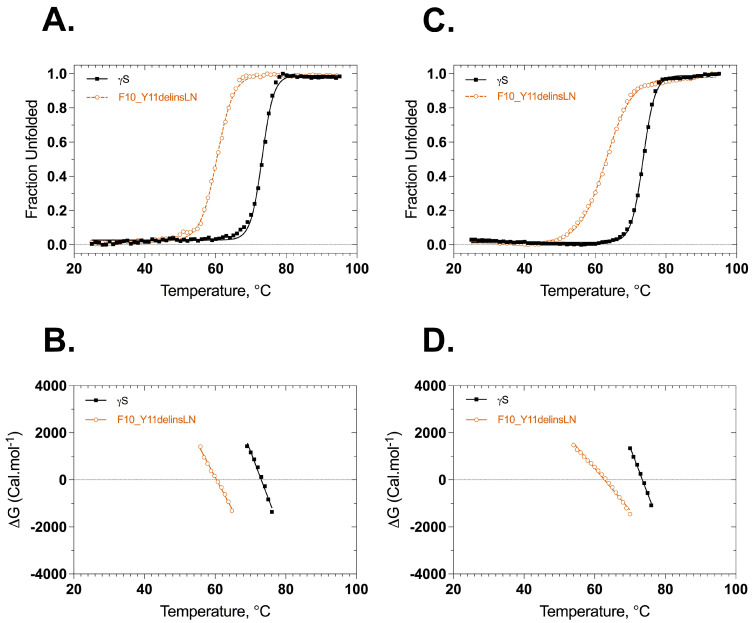
Thermal denaturation profiles of wild-type (black closed squares) and F10_Y11delinsLN mutant (brown open circles) human γS-crystallin from circular dichroism and fluorescence spectroscopy. (**A**) Unfolding curves according to CD. (**B**) ΔG Values calculated from the above plots (**A**) as a function of temperature. ΔG values were calculated from the equation ΔG = −RTLnK. (**C**) Thermal unfolding curves monitored by tryptophan emission. λ_exc_: 285 nm. (**D**) ΔG values calculated from the above plots (**C**) as a function of temperature. Solid lines represent the two-state fit.

Stabilities of wild-type and F10_Y11delinsLN γS-crystallins were assessed under thermal and GuHCl-induced stress. Thermal stability was assessed by heating the protein from 25 °C to 95 °C and monitoring the ellipticity at 218 nm or the 355/330 nm ratio of tryptophan fluorescence as a function of temperature. The CD thermal denaturation curves show that both the wild-type and F10_Y11delinsLN proteins underwent two-state transitions but with different onset points, slopes, and midpoint T_m_ values (Figure 3A,B). The mutant unfolding curve shows an onset point around 50 °C with a T_m_ of 60.4 °C and complete denaturation around 70 °C. In contrast, the wild-type curve shows an onset around 64 °C with a T_m_ of 73.1 °C and complete denaturation at around 80 °C. The overall T_m_ difference between wild type and mutant is 12.7 °C. The thermal denaturation analysis using the tryptophan emission fluorescence ratio (355/330) shows similar results (Figure 3C,D) with a transition midpoint of 62.8 °C for the F10_Y11delinsLN mutant and 73.5 °C for the wild-type γS-crystallin proteins. The overall ΔT_m_ determined by this method is 10.7 °C. However, while the denaturation curve of the mutant does not show a distinct intermediate stage, it does show a more gradual upslope than the wild-type curve, suggesting that the protein fold of the N-terminal domain might be opening up and exposing the normally buried tryptophans to the hydrophilic surface earlier than the C terminal domain, or either domain in the wild type. Similarly, while the wild-type and mutant both exhibited single first derivate maxima, which further confirms that they undergo two-state transitions (Figure S3A), the derivative of the fluorescence ratio curve of the mutant shows a shoulder at lower temperatures. Thus, in contrast to the wild-type first derivative, which is sharp and symmetrical, the mutant shows a broad asymmetric first derivative peak spanning from 40 °C to 76 °C, consistent with destabilization of the mutant N-terminal domain at lower temperatures.

ΔG values were calculated from the ellipticities at 218 nm or fluorescence ratio (350 nm/330 nm) at various temperatures using the equation ΔG° = −RTlnK, where R is the gas constant 1.987 kcal K^−1^ mol^−1^, and is plotted against the temperatures as shown in Figure 3B,D. The ΔG plots derived from the ellipticities are almost parallel to each other, with the F10_Y11delinsLN mutant ΔG plot crossing 0 at 60.4 °C and that for the wild type crossing 0 at approximately 73.1 °C (Figure 3B, Table 1). In contrast, the ΔG plots derived from the fluorescence ratio show a greater difference in slope, corresponding to the more gradual upslope of the fluorescence ratio of the mutant, with the F10_Y11delinsLN mutant ΔG plot crossing 0 at 62.8 °C and that for the wild type crossing 0 at approximately 73.5 °C (Figure 3D, Table 1). There is no discernable break in the ΔG plot for the F10_Y11delinsLN mutant, as would be expected for a three-state denaturation process. The ΔH values calculated from these plots for the wild type are 137.3 and 137.4 kcal mol^−1^ and for the mutant are 95.2 and 59.6 kcal mol^−1^ from CD and fluorescence-based unfolding methods, respectively. The Van’t hoff plots also followed similar trends, although there was a slight indication of a three-step denaturation process for the F10_Y11delinsLN mutant (Figure S3B,C). Overall, thermal denaturation as assessed using both the CD and tryptophan fluorescence emission methods confirms that the mutant is structurally less stable than the wild-type crystallin.

The thermal stability was also estimated by measuring the secondary structure of the wild type and F10_Y11delinsLN mutant at the transition midpoint of the mutant (61 °C) with different incubation times from 200 to 900 s. The spectra of the wild type were similar at all time points (Figure S3D), but the mutant shows a gradual loss of molar ellipticity intensity at 218 and 206 nm with increasing incubation times consistent with loss of its secondary structure (Figure S3E), the loss of structure occurring incrementally between 200 and 800–900 s. The 218 and 206 nm bands in the mutant display a decrease of 677 units (from −5920 to −5243) and 603 units (from −3960 to −3357) in the 400–500 s time range, while the wild type shows a difference of only 8 and 4 units at 218 and 206 nm, respectively. The loss of its secondary structure further confirms that the mutant is less stable and thus is more susceptible to denaturation at high temperatures than the wild type, and that this process occurs globally and gradually with increasing exposure times.

### 2.5. Stability of Wild-Type and F10_Y11delinsLN Mutant γS-Crystallin under Chemical Stress

**Figure 4:**
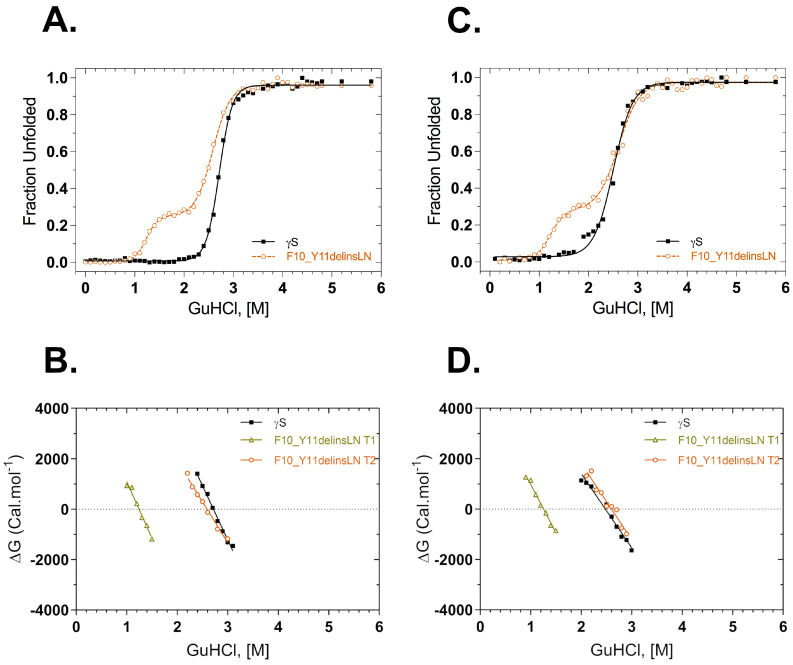
GuHCl equilibrium unfolding and refolding of wild-type (black squares) and F10_Y11delinsLN mutant (brown circles) human γS-crystallin probed with tryptophan fluorescence emission. (**A**) GuHCl unfolding curves. λ_exc_: 295 nm. (**B**) ΔG values calculated from the above GuHCl denaturation curves (**A**) as a function of GuHCl concentration. (**C**) GuHCl refolding curves. λ_exc_: 295 nm; (**D**) ΔG values calculated from the above refolding curves (**C**) as a function of GuHCl concentration. Solid lines represent the two-state fit for the equilibrium unfolding and refolding. T1: Transition-1; T2: Transition-2.

Thermodynamic stability was also assessed under GuHCl chemical-induced stress. Fluorescence intensity at 355/330 nm was used to monitor structural changes in the native environment and under increasing concentrations of GuHCl (Figure 4A). The GuHCl unfolding profile of the F10_Y11delinsLN mutant differs from that of the wild type, exhibiting a three-state transition with a clear destabilized N-terminal intermediate populated around 1.25 M GuHCl. In contrast, the wild type shows a sharp two-state transition. The C-terminal domain transitions of the wild type and F10_Y11delinsLN mutant are similar, occurring at around 2.72 and 2.59 M GuHCl, respectively. ΔG values are shown as a function of GuHCl in Figure 4B. ΔG plots for the second transition were similar in both the proteins, but the mutant shows an additional ΔG value from the transition at 1.25 M. ΔG values calculated for the first and second transition of the mutant are 5.6 kcal mol^−1^ and 8.4 kcal mol^−1^, respectively, while for the wild type transition it is 11.7 kcal mol^−1^. The refolding curves and their derived ΔG plots show similar trends (Figure 4C,D) in that the curve for the mutant shows two clear transitions at 1.26 M and 2.60 M, while the curve for wild-type γS-crystallin exhibits only a single transition at around 2.48 M, although there is some scatter at the beginning of the transition (Figure 4C). Some small differences were seen between the unfolding and refolding parameters of the wild type and mutant (Table 2).

### 2.6. Aggregation of Wild-Type and F10_Y11delinsLN Mutant γS-Crystallin and Interaction with αA-Crystallin

Wild-type and F10_Y11delinsLN mutant γS-Crystallin interactions with αA-crystallin under benign and stressed conditions were also investigated. Purified αA-crystallin elutes at 500 kD (9–13 mL) on size exclusion chromatography (Figure S4A). Its far-UV CD spectrum displays a typical 218 nm β-sheet conformation (Figure S4B), its near-UV CD spectrum shows negative ellipticity (Figure S4C), and its tryptophan emission spectrum shows an emission maximum around 339 nm (Figure S4D), all consistent with published results [16].

**Figure 5:**
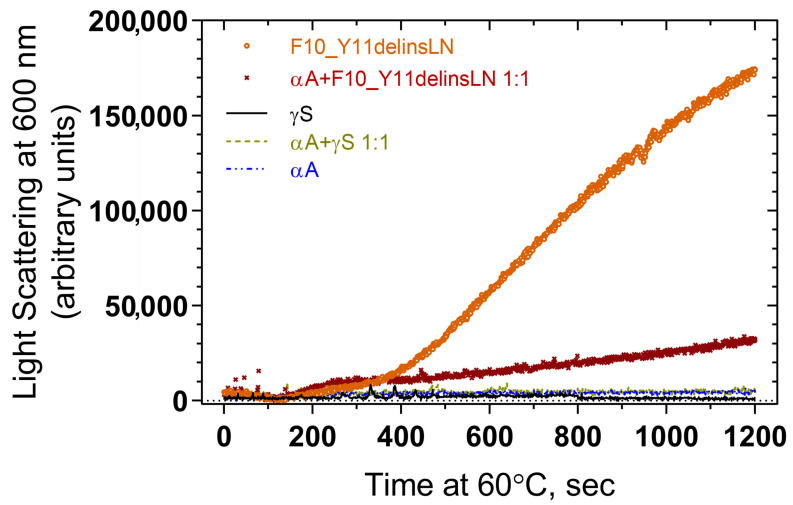
Light scattering measurement of thermal aggregation profiles of wild-type (black line) and F10_Y11delinsLN mutant (brown circles) human γS-crystallin, and of a 1:1 molar ratio of each (light green dotted line and maroon Xs, respectively) with αA-crystallin (blue broken line).

Association of αA-crystallin with wild-type and mutant F10_Y11delinsLN γS-crystallin under benign and thermal stress conditions were tested by heating the protein samples at 60 °C and checking for light scattering by particles at 600 nm. Wild-type γS- and αA-crystallins show little light scattering over the entire 1200 s exposure, while the mutant alone shows increasing levels of light scattering particles beginning at 400 s (Figure 5). A 1:1 molar ratio mixture of αA-crystallin and F10_Y11delinsLN mutant γS-crystallin displays significantly less light scattering and a slower increase in light scattering compared to the F10_Y11delinsLN mutant γS-crystallin alone, indicating that αA-crystallin is associating with the mutant as it begins to denature under thermal stress and preventing it from forming HMW aggregates or other light scattering particles.

**Figure 6:**
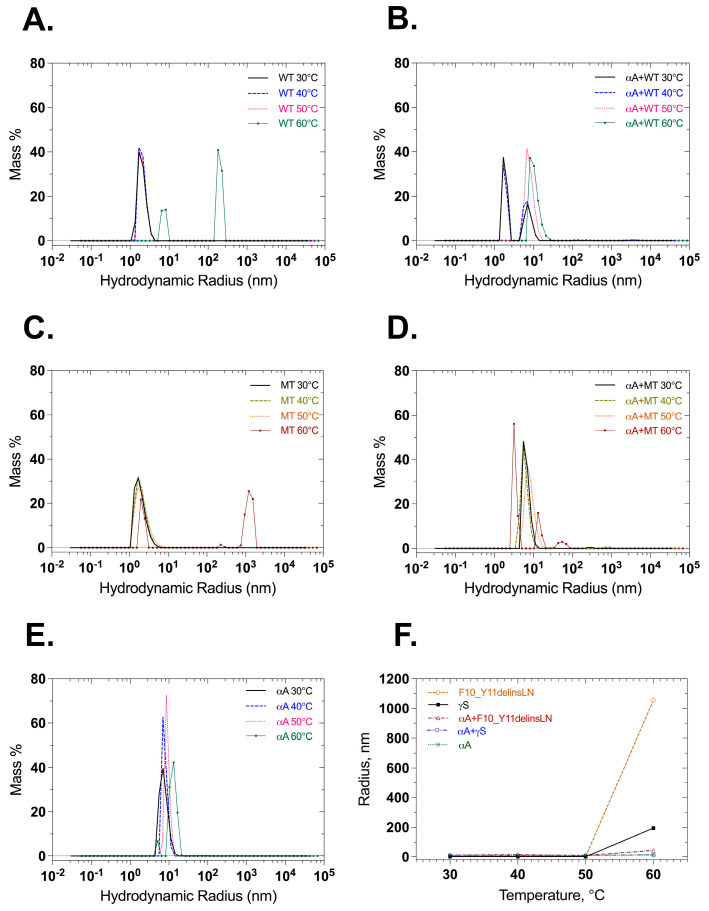
Particle size and distribution measured at different temperatures by dynamic light scattering. (**A**) Wild-type γS-crystallin, (**B**), αA-crystallin + wild-type γS-crystallin 1:1 molar ratio, (**C**) F10_Y11delinsLN mutant γS-crystallin, (**D**) αA-crystallin + F10_Y11delinsLN mutant γS-crystallin 1:1 molar ratio. (**E**) αA-crystallin and (**F**) hydrodynamic radii shown as a function of temperatures: 30, 40, 50, and 60 °C. WT: wild-type γS-crystallin, MT: F10_Y11delinsLN Mutant γS-Crystallin, αA: αA-Crystallin.

Dynamic light scattering was also used to check the particle size of wild-type and mutant γ-crystallin proteins with and without αA-crystallin under benign conditions and thermal stress. Wild-type γS-crystallin maintains small radius equivalents, below 20 nm through 50 °C, and shows particles with radius equivalents of 90 and 200 nm, at 60 °C. αA-Crystallin maintains a radius equivalent below 20 nm, as does a 1:1 mixture of wild-type γS- and αA-crystallin (Figure 6A,B,E,F). Mutant F10_Y11delinsLN γS-crystallin maintains a small radius until 50 °C but shows particles of 1063 nm radius after heating to 60 °C. In contrast, a 1:1 molar ratio mixture of mutant F10_Y11delinsLN γS-crystallin and αA-crystallin shows particles of 13.3 nm radius throughout the temperature range (Figure 6C,D,F), confirming the ability of αA-crystallin to bind mutant F10_Y11delinsLN γS-crystallin as it begins to denature at 60 °C, preventing it from forming large light-scattering particles. When the intensity distribution is examined, the mutant displays two peaks between 5 and 100 nm in the 30–50 °C range (Figure S5C). The wild type also displays two populations, but the level of intensity emitted in the 100 nm range is comparatively less than the mutant (Figure S5A). At 60 °C, wild-type and F10_Y11delinsLN mutant γS-crystallins both show a single intensity distribution around the 100–200 and 1000 nm range, respectively, and this disappears completely in the presence of αA-crystallin (Figure S5B,D) indicating association of αA-crystallin with wild-type or F10_Y11delinsLN mutant γS-crystallin around 60 °C with consequent reduction of higher-level aggregation. αA-Crystallin alone shows only peaks in the 10–100 nm range throughout the temperature range (Figure S5E).

**Figure 7:**
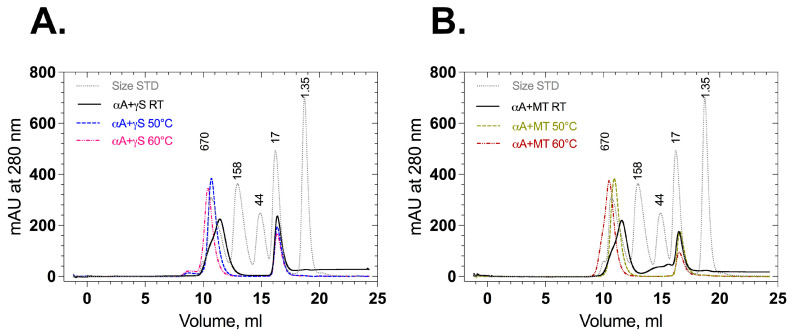
Size exclusion chromatography profiles of wild-type and F10_Y11delinsLN mutant human γS-crystallin in the presence of αA-crystallin. (**A**) Association of αA-crystallin with wild-type and (**B**) F10_Y11delinsLN mutant γS-crystallin at an 8:1 molar ratio at room temperature and stress conditions. (50 °C or 60 °C for 50 min as indicated).

Association of αA-crystallin with wild-type and mutant γS-crystallin in their native and stressed states was also checked by analytical size exclusion chromatography. Mixed wild-type γS-crystallin and αA-crystallin displayed two elution peaks around 9–13 mL (500 kDa) and 16–18 mL (20 kDa) (Figure 7A), as did mixed F10_Y11delinsLN γS-crystallin and αA-crystallin (Figure 7B). No additional peaks corresponding to higher aggregates were observed. When the proteins (αA-crystallin + wild type or αA-crystallin + mutant F10_Y11delinsLN) were stressed and loaded onto size exclusion chromatography columns, the peak around 9–13 mL (500 kDa) was sharp and shifted slightly to its left. Mass spectrometry analysis was performed to identify the proteins in each peak (Table S1). In room-temperature-incubated (native) samples (αA-crystallin + wild type or αA-crystallin + mutant F10_Y11delinsLN), the peak at around 9–13 mL contains pure αA-crystallin and the peak at around 15–18 mL displayed either wild-type or mutant γS-crystallin (Supplementary Table S1A,B). In αA-crystallin + wild-type γS-crystallin samples stressed at 50 °C, the peak at around 9–13 mL had a mass similar to that of αA-crystallin (20,729.7 ± 1 Da, Table S1C). However, in the αA-crystallin + mutant F10_Y11delinsLN samples stressed at 50 °C, the peak at around 9–13 mL had a mass similar to that of αA-crystallin peak (20,729.7 ± 1 Da) along with F10_Y11delinsLN mutant γS-crystallin around 9.5 mL (Table S1D). In the αA-crystallin + wild-type sample stressed at 60 °C, the peak at around 9–13 mL still showed a mass consistent with that of αA-crystallin (20,729.7 ± 1 Da, Supplementary Table S1E), but the αA-crystallin + mutant F10_Y11delinsLN sample showed αA-crystallin and mutant F10_Y11delinsLN γS-crystallin in both the 9.5 and 10.5 mL fractions (Table S1F), indicating that the mutant alone is associating with αA-crystallin under thermal stress at 50 °C and 60 °C. In all cases, the fractions around 15–18 mL displayed either wild-type (20,870.4 ± 2 Da) (Table S1A,C,E) or mutant mass (20,787.5 ± 1 Da) (Table S1B,D,F), depending on the samples loaded. Close inspection of SEC shows that the 15–18 mL peak height of wild-type γS-crystallin is almost the same for all temperatures, but it is decreased to half of its height in the F10_Y11delinsLN mutant γS-crystallin sample stressed at 60 °C compared to the room-temperature sample, consistent with the appearance of the F10_Y11delinsLN mutant γS-crystallin in the exclusion peak. Thus, the F10_Y11delinsLN mutant but not wild-type γS-crystallin is bound by αA-crystallin under stressed (50 °C and 60 °C) conditions, consistent with the DLS results.

### 2.7. Effects of Reducing Agents on Aggregation of Wild-Type and F10_Y11delinsLN Mutant γS-Crystallin

**Figure 8:**
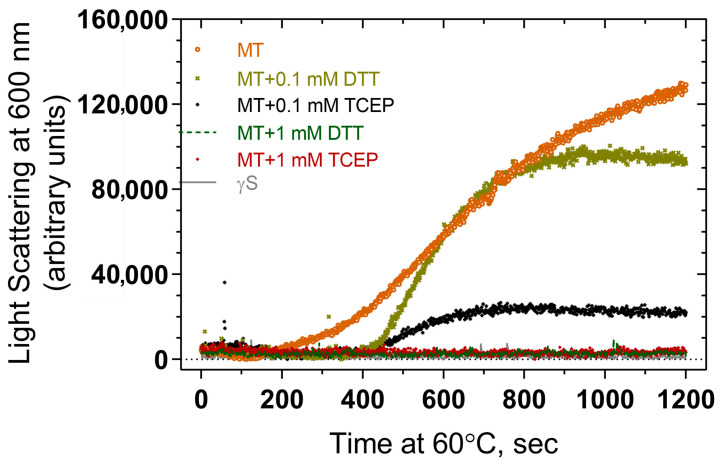
Effect of reducing agents (DTT and TCEP) on thermal-induced aggregation of F10_Y11delinsLN mutant human γS-crystallin. MT: F10_Y11delinsLN mutant γS-crystallin.

The effects of reducing agents DTT and TCEP at various concentrations in suppressing thermal aggregation were examined using light scattering measurements. Both DTT and TCEP suppress aggregation to some extent compared to that seen in their absence, including the early linear phase of the curve and the later plateau region (Figure 8). While there is some variation in aggregation levels in the presence of the reducing agents, both 1 mM DTT and 1 mM TCEP suppress aggregation to background levels, suggesting that both agents can prevent aggregation, and the differences between curves at 0.1 mM could perhaps represent differing strengths of reducing power.

## 3. Discussion

The γ-crystallins have extremely rigid three-dimensional structures with tight packing [17]. Five aromatic pairs Y11-F16, Y50-F55, F99-F104, Y140-Y145, and Y21-Y33 are important for maintaining their Greek key motif structures and play a critical role in providing stability and compactness to the γ-crystallin protein. In the cataract-associated mutation F10_Y11delinsLN, F10 and Y11 in the first Greek key motif of the N-terminal domain are replaced with L10 and N11. This work investigates how these substitutions affect the structure and stability of the protein and the relationship of these effects to the cortical lamellar cataracts described in family members carrying this mutation [10].

**Figure 9:**
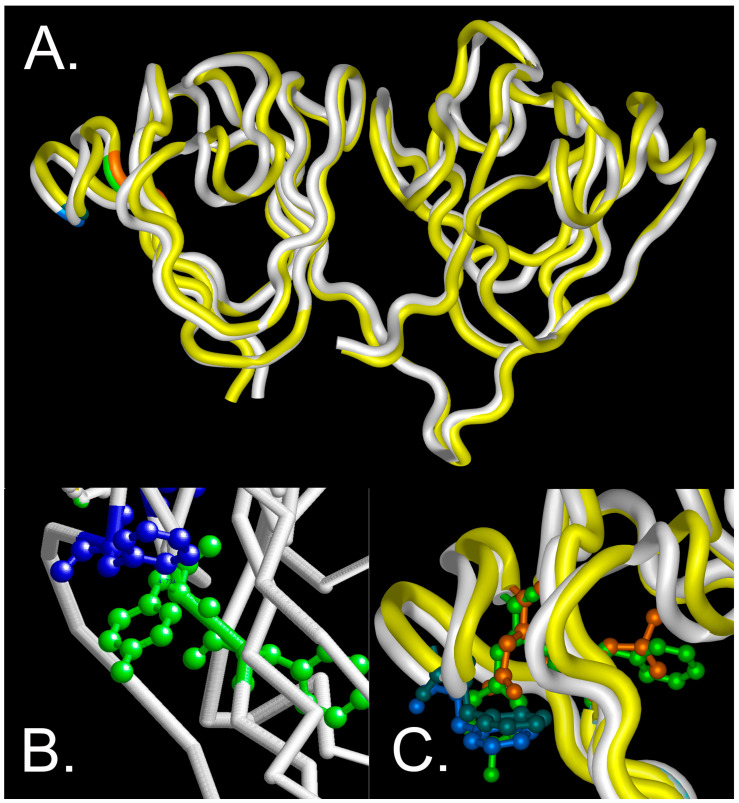
Molecular modeling of wild-type and F10_Y11delinsLN mutant γS-crystallin. (**A**) overview of the protein backbone of the wild-type (white) and F10_Y11delinsLN mutant (yellow) γS-crystallin. The carboxyl-terminal domain (right) shows essentially complete overlap, while the amino-terminal domain (left) shows slight distortion of the first and second Greek key motifs. The display is shown from the DNASTAR (Madison WI) Protein Analysis and Modeling module using tube rendering. F10 and Y11 in the wild type are shown in green, L10 and N11 in the mutant γS-crystallin are shown in orange, and F16 in both is shown in blue. (**B**) Close-up view of the wild-type structure backbone as depicted in RasMol v. 2.7.4.2 (Bellport, NY, USA) with F10 and Y11 shown in green ball and stick mode and F16 and the inter-residue distance to Y11 shown in blue. (**C**) Close-up DNASTAR tube rendering of the overlaid wild-type and F10_Y11delinsLN mutant γS-crystallin proteins with F10 and Y11 in the wild type shown in green ball and stick mode, L10 and N11 in the mutant γS-crystallin shown in orange, F16 shown in bright blue in the wild type and blue green in the F10_Y11delinsLN mutant γS-crystallin.

The total accessible surface area that is occupied by the changed amino acids is decreased from 218 to 180 (Å^2^) (F10 to L10) and 229 to 158 (Å^2^) (Y11 to N11) [18], suggesting that alterations in the size of these residues is not a major factor in destabilization of the Greek key motif. However, the π interactions around the site might be altered with subsequent destabilization of the protein, since the substitutions do not significantly alter the backbone conformation as estimated by CD under physiological conditions. This is consistent with molecular modeling of the wild-type and F10_Y11delinsLN mutant protein, which shows that the protein fold is essentially maintained in the carboxyl domain and only slightly distorted in the amino terminal domain (Figure 9A). In the wild-type model, the distance between Y11 and F16 is estimated to be 4.73 Å (Figure 9B), consistent with stabilization in a perpendicular orientation [8]. The indel does alter the tryptophan micro-environment (Figure 9C) consistent with the changed near-UV cd and fluorescence spectra (Figure 1B,C). Consistent with this observation, increased exposure of hydrophobic residues to the surface of the mutant protein even under physiological conditions was confirmed via both bis-ANS and Nile Red fluorescence, similar to the results obtained with the G18V γS-crystallin and Y46D γC-crystallin mutations [11,13], and showing increased ANS binding to previously buried residues in the N-terminal domain and the nearby interdomain interface [19]. However, in G18V γS-crystallin, this difference increased significantly upon exposure to 1 M GuHCl [13] and the Y46D γC-crystallin mutant shows a greater difference in binding between bis-ANS than Nile Red at low concentrations, in contrast to the F10_Y11delinsLN γC-crystallin, which shows significant binding with both agents even at low concentrations.

While the increased exposure of hydrophobic residues to the protein surface indicates an opening of the protein fold, the circular dichroism and tryptophan fluorescence studies indicate that the mutations have only a minor effect on the overall protein structure under benign conditions approximating physiological exposures. However, the decreased stability is still present and simply becomes more obvious under stress, which accelerates the structural changes. Thus, while denaturation and aggregation would be much slower under physiological conditions, proteins in the lens fiber cells do not turn over, and even minor instability will manifest itself in structural changes over the months of prenatal development and especially the months and years of childhood and adult exposure to environmental stress. While the lens has large amounts of α-crystallins that act as chaperones to bind unstable and partially denatured proteins as well as high concentrations of reducing agents such as glutathione, these are eventually overcome, resulting in clinical cataract, either by scattering light directly or through damaging the lens cells and disrupting the lens microarchitecture [20]. In general, one would expect an inverse correlation between the decrease in stability of a crystallin and the length of time required for denaturation and aggregation of the mutant protein under physiological conditions. In contrast, the spatial expression pattern of a mutant crystallin is better correlated with the location of resultant cataracts, although this correlation is imprecise. γS-crystallin is highly expressed in the equatorial epithelia, the cortical fiber cells and the nuclear fiber cells [21], which correlates well with CRYGS mutations most frequently causing cortical, lamellar, sutural or nuclear cataracts [22]. The cortical cataracts seen in this case are consistent with that pattern.

The mutations do destabilize γS-crystallin significantly under both thermal and chemical stress. In combination, the mutations decrease the overall stability to 5.0 kcal mol^−1^ and lower the transition midpoint to 12.7 °C in thermal unfolding experiments monitored by CD. The energy change observed from fluorescence-based thermal unfolding data was similar, 4.3 kcal mol^−1^. There is a slight difference in ΔH values and slopes in the unfolding procedures, although this is probably due to differences in the methods with regard to holding times and monitoring [13]. The mutant appears to undergo a primarily two-state transition under thermal stress, although the shoulder seen in the tracing suggests that the N-terminal domain begins to destabilize a bit earlier when subjected to thermal stress. In contrast, the mutant shows a clear N-terminal intermediate transition under GuHCl-induced stress, during which it exhibits an intermediate around 1.25 M and the c-terminal transition is lowered to 2.59 M compared to 2.72 M in the wild type. This probably relates to the greater sensitivity of the fluorescence-based curves to small changes in the neighborhood of tryptophan and tyrosine residues while the CD measurements reflect the overall protein fold, similar to results seen with the G18V CRYGS and Y46D CRYGC mutations [11,13].

The role of aromatic pair interactions on the stability of the human γD-crystallin protein has been well studied. N-terminal domain substitutions affect the stability of the protein by shifting transitions of that domain to lower GuHCl concentration while the C-terminal domain transitions remained relatively unaffected. In addition, Greek key pairs tend to contribute more to the thermal and thermodynamic stability of the domain than non-Greek key pairs [23]. The Y11-F16 aromatic pair in human γS-crystallin is homologous to the Y7-F12 pair in human γD-crystallin. When the Y7 residue of γD-crystallin is changed to alanine, γD-crystallin shows a two-state transition in thermal unfolding with an 8.9 °C decrease in its T_m_ [23], similar to the present study, in which F10_Y11delinsLN γS-crystallin shows a decrease of 12.7 °C. An alanine substitution at Y6 in γD-crystallin resulted in a three-state transition under GuHCl treatment, with a concentration midpoint of 1.12 M GuHCl [23] and a similar trend was also observed in the current study, in which the substitution caused a three-state transition with an intermediate around 1.25 M, which is consistent with Greek key pair domain substitutions showing severe effects. Phenyl rings in aromatic pairs interact optimally at a distance of 4.5–7 Å, aligning perpendicular or parallel to each other, and contributing a free energy of 0.5–1.3 kcal mol^−1^ to overall protein stability [8,9], supporting the importance of aromatic pairs in stabilizing the protein. Substitution of two amino acids rather than a single change also contributes to the destabilization being more severe than that seen in the Y7-F12 change in γD-crystallin. Here, not only does the F10_Y11delinsLN mutation studied disturb the first Greek key aromatic pair interaction, but the adjacent F10 to L10 change also contributes to destabilization by further distorting the Greek key motif. Significant and even minor distortion of the protein fold of the Greek key motif can also lead to significant destabilization as shown by the results of Ma et al. [13], in which even the conservative G18V CRYGS change in a critical part of the motif structure reduced the T_m_ by 9.5 °C, similar to the results seen here. An even greater destabilization of the second Greek key motif was seen in the Y46D γC-crystallin mutant, which showed a ΔT_m_ of −23 and 24.4 °C with CD and fluorescence, respectively, for the native to intermediate form and −9.9 and 9.3 °C for the intermediate to unfolded species corresponding to Δ(ΔG) values of −11 and −4.7 kcal mol^−1^ for the CD curves and −9.1 and −3.5 kcal mol^−1^ for the fluorescence curves. Similarly, GuHCl unfolding curves yielded ΔG values of 3.2 and 6.2 kcal mol^−1^ for the intermediate and unfolded Y46D mutant species, respectively vs. 10.5 for the wild type [11]. 

While both intrinsic fluorescence and ANS and Nile Red binding studies demonstrate that the surface hydrophobicity of the F10_Y11delinsLN mutant γS-crystallin is increased, size exclusion chromatography of αA-crystallin alone and mixed with wild-type and F10_Y11delinsLN mutant γS-crystallins shows that αA-crystallin does not bind either the wild-type or mutant protein to any appreciable extent under physiological conditions. However, as the F10_Y11delinsLN mutant γS-crystallin is heated at 60° both light scattering at 600 nm and dynamic light scattering show that it forms HMW aggregates, and that this process is prevented by a 1:1 molar ratio of αA-crystallin. These studies show that the F10_Y11delinsLN mutant γS-crystallin does not escape binding by αA-crystallin as some rapidly denaturing proteins do [24,25]. In contrast, analytical size exclusion chromatography results confirmed that αA-crystallin is not associated with F10_Y11delinsLN mutant and wild-type γS-crystallin at room temperature under benign conditions. This differs from the G18V mutant, which appears to associated with αA-crystallin by NMR analysis [26], although the NMR technology might be more sensitive than size exclusion chromatography, which would only show stable binding. The F10_Y11delinsLN mutant aggregates and scatters light upon heating at 60 °C, and both DTT and TCEP suppress thermal aggregation by between 2- and 4-fold, differing from the Y46D γC-crystallin mutant, which does not show inhibition of aggregation by either DTT or TCEP [11]. This probably relates to the involvement of disulfide bonds in aggregation of the F10_Y11delinsLN γS-crystallin but not the Y46D γC-crystallin protein. Few other studies have focused on the structural characterization of mutants in γS-crystallin. The D26G [27] and G57W [28] mutations show only minor effects on stability. In contrast, G18V [13,29], S39C [7], and V42M [30] show somewhat more severe changes, and the present work places the F10_Y11delinsLN mutation in the second group, showing a relatively normal protein fold under benign conditions, but a marked decrease in stability under thermal or chemical stress.

## 4. Materials and Methods

### 4.1. Cloning and Site-Directed Mutagenesis

Human γS-crystallin cDNA was cloned into the *Nde*I*/Xho*I sites of pET-20b (+) (Novagen, Burlington, MA, USA) as described earlier [13]. To generate the F10_Y11delinsLN double mutant clone, the wild-type clone was amplified with the following primers F-5′ CAAGATTACTTTAAATGAAGACAAAAATTTTCAAGG 3′, R-5′ GAAAATTTTTGTCTTCATTTAAAGTAATCTTGGTTCC 3′. Methylated wild-type DNA was eliminated via digestion with *Dpn*I, and the remaining plasmid was transformed into *E. coli* DH5α. Human αA-crystallin cDNA was cloned into the *Nde*I*/Xho*I sites of pET-21a (+) (Novagen) using a reverse primer harboring a CACCACCACCACCACCAC (6X his tag) sequence just before the stop codon. Plasmids were isolated from the transformed *E. coli* colonies and bidirectionally sequenced to confirm the appropriate base pair changes and the absence of nonspecific mutations.

### 4.2. Protein Overexpression

Proteins were overexpressed and purified as described earlier [13,30]. BL21(DE3)pLysS cells were used to express the wild-type and mutant proteins. Single colonies containing the respective clones were inoculated into 12 mL of LB broth containing 50 μg/mL ampicillin and 34 μg/mL chloramphenicol and grown for 12 h at 37 °C with 225 rpm shaking. Then, 10 mL of this stationary phase culture was transferred to 1 L LB broth with the same concentration of antibiotics. The inoculated broths were grown at 37 °C with 225 rpm shaking until they reach an OD_600_ of 0.6–0.8. The cultures with the F10_Y11delinsLN bacterial clone were adjusted to 0.25 mM IPTG and grown at 25 °C for an additional 6 h, whereas the cultures with wild-type γS and αA clones were induced with a final concentration of 1 mM IPTG and grown at 37 °C for an additional 4 h. Induced cells were pelleted via centrifugation at 6000× *g* for 10 min at 4 °C and frozen at −80 °C until use.

### 4.3. γS-Crystallin Purification

Pellets containing overexpressed proteins were resuspended in 50 mM Tris-Cl pH 7.3, 100 mM NaCl, 1 mM EDTA, 1 mM DTT, 0.25 mM TCEP, and a protease inhibitor mix (Roche Diagnostics; Catalog #11836153001). A final lysozyme concentration of 0.3 mg/mL and a final DNase concentration of 7.5 µg/mL were maintained in the suspensions. Cell suspensions were freeze–thawed for 3 cycles, each cycle consisting of 5 min incubation on dry ice and 5 min incubation in a 37 °C water bath. The freeze–thawed cell suspensions were further sonicated at 4 °C and 20% amplitude with an Omni international Sonic Ruptor-400 (Kennesaw, GA, USA) using 9 cycles for the mutant, with each cycle comprising a 5 s pulse on and a 55 s pulse off, and using 6 cycles for the wild type, with each cycle comprising a 10 s pulse on 50 s pulse off. The cell lysate was centrifuged at 30,000× *g* for 20 min at 4 °C, and the supernatant was loaded into an 8 mL 10,000 MWCO dialysis cassette (Pierce Biotechnology, Rockford, IL, USA) and dialyzed against 2 L of buffer A (50 mM CH_3_COONa, 1 mM EDTA, 1 mM DTT, 50 μM TCEP, pH 5.4) for 6 h.

Soluble extracts were purified at room temperature with ion-exchange and size-exclusion chromatography using an NGC™ Chromatography System (Bio-Rad, Hercules, CA, USA). The dialyzed supernatant was centrifuged at 30,000× *g* for 20 min at 4 °C, and samples were loaded onto a 5 mL HiTrap SP FF (GE Healthcare, Chicago, IL, USA; Catalog #17-5157-01) column equilibrated with buffer A at a flow rate of 1.0 mL/min. The column was washed with five column volumes of buffer A. Then, a linear gradient of buffer B (50 mM CH_3_COONa, 1 mM EDTA, 1 mM DTT, 50 μM TCEP, pH 5.4, and 1 M NaCl) was applied and fractions were collected.

### 4.4. αA-Crystallin Purification

αA-Crystallin was also purified using a modification of the above-described method. The lysis buffer used in purifying αA crystallin was 50 mM KH_2_PO_4_/K_2_HPO_4_ pH 8.0, 300 mM KCl, 1 mM EDTA (buffer C) with a protease inhibitor mix (Roche Diagnostics, Basel, Switzerland; Catalog #11836153001). Sonication was carried out as described above for the wild-type protein (6 cycles, each cycle comprising a 10 s pulse on and a 50 s pulse off), and the centrifuged cell lysate was loaded onto a HisTrap HP (GE Healthcare; Catalog #17524801) column equilibrated with buffer C at a flow rate of 1.0 mL/min. Five column volumes of buffer C were passed through the column to wash away the unbound proteins completely, then, a linear gradient of buffer D (50 mM KH_2_PO_4_/K_2_HPO_4_ pH 8.0, 300 mM KCl, 1 mM EDTA, and 0.5 M imidazole) was applied and fractions were collected.

The fractions containing the proteins of interest were monitored with absorbance at 280 nm and SDS-PAGE on 12% polyacrylamide gels, and were pooled and concentrated to 10–15 mg/mL. Wild-type and mutant γS-crystallin protein pools were separately loaded onto a HiPrep™ 16/60 Sephacryl^®^ S-100 HR column (GE Healthcare; Catalog #17116501) previously equilibrated with SEC buffer (50 mM NaH_2_PO_4_/Na_2_HPO_4_ (pH 7.3), 0.15 M NaCl, 1 mM EDTA, 1 mM DTT, and 50 μM TCEP) at a flow rate of 0.5 mL/min. Pooled wild-type αA-crystallin was injected on to a HiPrep™ 16/60 Sephacryl^®^ S-300 HR column (GE Healthcare; Catalog #17116701) equilibrated with SEC buffer at a flow rate of 0.5 mL/min. The columns were pre-calibrated with five standards: thyroglobulin, γ-globulin, ovalbumin, myoglobin, and vitamin B12 (Bio Rad gel filtration standard; Catalog #1511901). The locations of recombinant proteins in column fractions were monitored using absorbance at 280 nm and by SDS-PAGE on 12% polyacrylamide gels. The purity of the protein was assessed and confirmed by the presence of a single band in SDS PAGE and then estimating the mass by using electrospray ionization mass spectrometry. Two independent preparations for each batch of αA, γS wild-types, and mutant proteins gave an average mass of 20,730.32 ± 0.5, 20,873.18 ± 0.5, and 20,789.76 ± 0.5 Da, respectively, in close agreement with the predicted monomer masses.

### 4.5. Liquid Chromatography/Mass Spectrometry (LC/MS)

Mass spectrometry data of 0.05 mg/mL proteins samples (in 50 mM Tris-Cl (pH 7.3), 150 mM NaCl, and 50 µM TCEP buffer) were acquired on an Agilent 6130 Quadrupole LC/MS System, (Agilent Technologies, Inc., Santa Clara, CA, USA) equipped with electrospray source, operated in positive-ion mode. Separation was performed on a 300SB-C3 Poroshell column (2.1 mm × 75 mm; particle size 5 μm). The analytes were eluted at a flow rate of 1 mL/min with a 5–100% organic gradient over 5 min and holding organic for 1 min. Mobile phase A contained 5% acetic acid in water, and mobile phase B was acetonitrile. Data acquisition and data analysis and deconvolution of mass spectra were performed using OpenLab ChemStation Edition software (version C.01.05).

### 4.6. Circular Dichroism (CD) Measurements

For determining secondary structure, 0.2 mg/mL protein samples in 10 mM NaH_2_PO_4_/Na_2_HPO_4_ (pH 7.3) were run between 260 and 185 nm at 25 °C in a 1 mm pathlength cuvette using a J-1500 (Jasco, Easton, MD, USA) circular dichroism instrument. For near-UV CD spectra, 1.0 mg/mL samples in 50 mM NaH_2_PO_4_/Na_2_HPO_4_ (pH 7.3), 150 mM NaCl, and 50 μM TCEP were analyzed between 350 and 250 nm at 25 °C in a 1 cm pathlength cuvette. A bandwidth of 1 nm, scanning speed of 20 nm/min for far-UV CD and 50 nm/min for near-UV CD and a digital integration time of 4 s at each point were maintained. At least five independent runs were measured and averaged, and spectra for the corresponding buffer for each protein were subtracted. For determining the secondary structure of wild-type and mutant proteins under GuHCl-induced stress, 0.15 mg/mL protein samples in 10 mM NaH_2_PO_4_/Na_2_HPO_4_ (pH 7.3) were incubated with varying concentrations of GuHCl (0–5 M) for 12–14 h at room temperature, and circular dichroism was measured between 260 and 185 nm at 25 °C in a 1 mm pathlength cuvette as described above. Three independent runs were measured and averaged, and blank spectra of the corresponding buffer were subtracted for each protein. Analysis of the CD spectra was performed as previously described by Fasman and coworkers [31,32].

Thermal denaturation experiments were performed by heating the test proteins from 25 to 95 °C with a ramping rate of 1 °C/min and collecting the 218 nm molar ellipticity using the same CD instrument as above. The protein concentration was 0.5 mg/mL in 50 mM NaH_2_PO_4_/Na_2_HPO_4_ (pH 7.3), 150 mM NaCl, and 50 μM TCEP. An equilibration time of 10 s at each temperature was maintained. Thermal denaturation curves were analyzed using GraphPad Prism 7 software according to the method of C.N. Pace [33]. The enthalpy change, ΔH, was calculated using the Van’t Hoff equation: d(lnK)/d(1/T) = −ΔH/R. The same protein concentrations, buffer strengths, and parameters maintained in recording the far-UV CD at 25 °C were used for the far-UV CD spectra at 61 °C. Samples were pre-equilibrated at 61 °C for 200 s prior to recording the spectra and were recorded at different time points ranging from 200 to 900 s as indicated.

### 4.7. Fluorescence Spectroscopic Measurements

For measuring tertiary structure using tryptophan fluorescence emission, 0.05 mg/mL protein samples in 52.5 mM NaH_2_PO_4_/Na_2_HPO_4_, 150 mM NaCl, 1 mM EDTA, 5 mM DT, and 50 μM TCEP were excited with 295 nm wavelength light and emissions were collected between 300 and 400 nm using a FluouroMax-4C Spectrofluorometer (Horiba Scientific, Edison, NJ, USA).

For determining the tertiary structure of wild-type and mutant proteins under GuHCl-induced stress, 0.1 mg/mL protein samples in 50 mM NaH_2_PO_4_/Na_2_HPO_4_ (pH 7.3), 150 mM NaCl, 1 mM EDTA, 5 mM DTT, and 50 μM TCEP were incubated with varying concentrations of GuHCl (0–5 M) for 12–14 h at room temperature and tryptophan emission was collected between 300 and 400 nm at 25 °C by exciting the samples at 295 nm. Three independent runs were measured and averaged, and the values for the corresponding buffer were subtracted as blanks for each protein.

Surface hydrophobicity and aggregation propensities were assessed using bis-ANS [14] and Nile Red [15]. For bis-ANS experiments, protein samples (0.33 mg/mL) in 66.7 mM NaH_2_PO_4_/Na_2_HPO_4_ (pH 7.3), 150 mM NaCl, 1 mM EDTA, 5 mM DTT, and 50 μM TCEP were incubated with a final concentration of dye ranging from 0.58 to 74.31 μM. For Nile Red experiments 0.1 mg/mL protein samples in 55 mM NaH_2_PO_4_/Na_2_HPO_4_ (pH 7.3), 150 mM NaCl, 1 mM EDTA, 5 mM DTT, and 50 μM TCEP were incubated with between 0.61 and 19.63 µM of the dye in the dark at room temperature for 30 min. Spectra for bis-ANS were recorded between 400 and 600 nm by exciting the samples at 390 nm and for Nile Red samples were recorded between 570 and 700 nm after excitation at 540 nm. Fluorescence intensities at 490 nm (bis-ANS) and 650 nm (Nile Red) were plotted against dye concentration.

For GuHCl unfolding experiments [34], samples with a final concentration of 0.1 mg/mL protein in 55 mM Tris-Cl (pH 7.3), 165 mM NaCl, 5 mM DTT, 1 mM EDTA, and 55 μM TCEP were incubated at room temperature for 14 h with stepwise increments of 1.0 M GuHCl until a final concentration of 4.8 M, sufficient to fully denature the proteins, was reached. Spectra were recorded at each step after equilibration. For refolding experiments, 1.0 mg of wild-type or mutant proteins were incubated in a final concentration of 5 M GuHCl, 64 mM Tris-Cl (pH 7.3), 191 mM NaCl, 5 mM DTT, 1 mM EDTA, and 64 μM TCEP at room temperature for 7 h, then diluted into a series of tubes containing refolding buffer (56 mM Tris-Cl (pH 7.3), 170 mM NaCl, 5 mM DTT, 1 mM EDTA, and 56 μM TCEP) until final concentrations of 0.1–4.8 M GuHCl (in 0.1 M GuHCl decrements) were reached and allowed to equilibrate at room temperature for 12–15 h. The same spectral conditions were used as for recording the tertiary structure with tryptophan emission, except for the excitation and emission slits.

GuHCl unfolding and refolding curves were analyzed by plotting the concentration of GuHCl for each sample versus the ratio of fluorescence intensities at 355 nm (maximum for the unfolded protein) and 330 nm (maximum for the native protein). The ratio of fluorescence intensities at these wavelengths was chosen for the analysis to allow simultaneous monitoring of changes in the native and unfolded maxima. Equilibria unfolding/refolding data were analyzed using GraphPad Prism 7 software, and ΔG° and m values were calculated using the method of C.N. Pace [33].

For determining thermal aggregation, a final concentration of 2.5 µM protein in 50 mM Tris-Cl (pH 7.3) and 150 mM NaCl was heated at 60 °C for 1200 s, and static (Rayleigh) light scattering at 600 nm was monitored using a spectrofluorometer for 1200 s with a 1 cm path length quartz cuvette. An equilibration time of 2 min at 60 °C prior recording the scattering was maintained. To check the effect of DTT and TCEP on thermal aggregation 2.5 µM protein in 50 mM Tris-Cl (pH 7.3), 150 mM NaCl with varying concentrations of TCEP and DTT was heated at 60 °C for 1200 s and light scattering at 600 nm was monitored.

All fluorescence experiments were carried out at 25 °C with 2.5 nm excitation and emission slits while maintaining 1 s integration time at each point. The excitation and emission slits used in the GuHCl unfolding experiments were 5 nm, and 10 nm excitation and emission slits were maintained in the refolding experiments.

### 4.8. Differential Scanning Fluorimetry Measurements

Thermal denaturation was also probed with tryptophan emission using a differential scanning fluorimeter (Prometheus NT.48, Nano Temper Technologies GmbH, Munich, Germany). The same protein concentrations and buffer strengths were maintained as for the CD thermal unfolding experiments. Samples were heated from 25 to 95 °C while being excited at 285 nm, and the 350/330 nm emission ratio was collected as a function of temperature.

### 4.9. Dynamic Light Scattering (DLS) Measurements

Wild-type and mutant protein particle sizes and distributions were assessed with dynamic light scattering using a DynaPro NanoStar dynamic light scattering instrument (Wyatt Technology, Santa Barbara, CA, USA). Protein solutions of 25 µM in 50 mM Tris-Cl (pH 7.3) and 150 mM NaCl buffer were illuminated using a 658 nm laser, and light scattered at a 90° angle was measured from 30 to 60 °C in 10 °C increments. A minimum of 20 acquisitions at each temperature step were recorded. Each protein solution was analyzed twice, and the average of all acquisitions is presented. A 1 °C/min ramping rate and an equilibration time of 2 min at each temperature was maintained before collecting acquisitions. To obtain the hydrodynamic radii the intensity autocorrelation functions were fitted by a regularization algorithm using Dynamics software version 7.10 (Wyatt Technology, Santa Barbara, CA, USA).

### 4.10. Analytical Size Exclusion Chromatography

Protein at a final concentration of 37.5 µM in 50 mM Tris-Cl (pH 7.3) and 150 mM NaCl was injected into a 24 mL ENrich SEC650 (Bio-Rad #780-1650) column equilibrated with 2 column volumes of 50 mM Tris-Cl (pH 7.3) and 100 mM NaCl. A flow rate of 0.5 mL/min was maintained, and the sample volume injected was not more than 1% of the column volume (240 μL). αA-crystallin was assessed by mixing αA-crystallin and F10_Y11delinsLN mutant γS-crystallin at an 8:1 molar ratio at room temperature and stress conditions before chromatography.

### 4.11. Molecular Modeling

The crystal structure of wild-type human γS-crystallin was taken from 7N36 in the RCSB PDB (https://www.rcsb.org, accessed on 30 May 2023) [35]. The modeling of the F10_Y11delinsLN mutantγS-crystallin protein was carried out using the Expasy Swiss-Model Workspace with the fully automated option using the 7N36.1.A structure as a template and default parameters (SWISS-MODEL Workspace—SIB Swiss Institute of Bioinformatics|Expasy). This provided 99% coverage (amino acids 5–178), 98.87% sequence identity and a GMQE of 0.88. The wild-type and mutant protein structures were overlaid using the Protein Analysis and Modeling module of DNASTAR version 17.1.1.120. Backbones structures and distances were viewed using the molecular graphics program RasMol version 2.7.4.2.

## 5. Conclusions

The F10_Y11delinsLN change relaxes the protein fold of the N-terminal Greek key motif under benign conditions, decreasing its stability under thermal or chemical stress, and also that of the N-terminal domain overall, leaving the C-terminal domain relatively unaltered initially. The partially denatured F10_Y11delinsLN γS-crystallin exists in a molten globule state and is bound by α-crystallin, eventually forming light-scattering HMW aggregates or possibly actually overwhelming the supply of α-crystallin in the lens and precipitating and damaging lens cells themselves. A combination of these processes then leads to the cortical lamellar cataract seen in the family with this mutation.

## Figures and Tables

**Table 1:** Thermal Denaturation Parameters of wild-type and mutant F10_Y11delinsLN γS-crystallin.

Proteins	T_m_ ^a^	ΔT_m_^b^	ΔS_m_^c^	ΔH_m_ ^d^	Δ(ΔG) ^e^
Wild type (CD curve) N↔U	73.1 ± 0.061		0.397 ± 0.021	137.3 ± 0.0012	
Mutant (CD curve) N↔U	60.4 ± 0.068	−12.7	0.285 ± 0.007	95.2 ± 0.0005	−5
			0.397 ± 0.021		
Wild type (FI ratio curve) N↔U	73.5 ± 0.036		0.396 ± 0.012	137.4 ± 0.0004	
Mutant (FI ratio curve) N↔U	62.8 ± 0.072	−10.7	0.178 ± 0.041	59.6 ± 0.0029	−4.3

^a^ Midpoint of thermal unfolding curve in °C. ^b^ Difference of T_m_ compared to wild type in °C. ^c^ Slope of (ΔG) versus T in (kcal mol^−1^ deg^−1^). ^d^ ΔH_m_ = [T_m_ (K) (ΔS_m_)] in (kcal mol^−1^). ^e^ Δ(ΔG) = [T_m_ ΔS_m_] where ΔS_m_ is the value for the wild-type protein in (kcal mol^−1^).

**Table 2:** Equilibrium Unfolding and refolding Parameters of wild type and F10_Y11delinsLN mutant γS-crystallin.

Protein	c_m_ ^a^	ΔG° (kcal mol^−1^) ^b^	m (kcal mol^−1^ M^−1^) ^c^
Wild type (UF)	2.72 ± 0.005	11.713 ± 0.490	4.310 ± 1.777
Mutant N↔I (UF)	1.25 ± 0.017	5.594 ± 0. 436	4.494 ± 0.345
Mutant I↔U (UF)	2.59 ± 0.008	8.409 ± 0.457	3.244 ± 0.179
Wild type (RF)	2.48 ± 0.011	7.318 ± 0.426	2.951 ± 0.168
Mutant N↔I (RF)	1.26 ± 0.039	4.805 ± 0.241	3.826 ± 0.198
Mutant I↔U (RF)	2.6 ± 0.020	7.873 ± 0.680	3.027 ± 0.271

^a^ Midpoint of GuHCl unfolding curve in M. ^b^ Free energy of unfolding in the absence of GuHCl in (kcal mol^−1^). ^c^ Slope of GuHCl versus FI ratio in (kcal mol^−1^ M^−1^).

## Data Availability

The data presented in this study are available in this article, the supplementary material.

## Supplementary Materials

The supporting information can be downloaded at: https://www.mdpi.com/article/10.3390/ijms241814332/s1.
