# Supplementary material for: Human γS-Crystallin Mutation F10_Y11delinsLN in the First Greek Key Pair Destabilizes and Impairs Tight Packing Causing Cortical Lamellar Cataract
Source: Int J Mol Sci. 2023 Sep 20;24(18):14332. doi: 10.3390/ijms241814332 (PMC10531703; doi:10.3390/ijms241814332)
Supplement: Supplementary file 1 [file ijms-24-14332-s001.zip › Supplemental Figure and Table Legends.pdf]

Figure S1. Far-UV CD spectra of wild type and F10_Y11delinsLN mutant human γS-crystallin in increasing concentrations of GuHCl. A) Wild type γS-crystallin. B) F10_Y11delinsLN mutant γS-crystallin. Scanning speed: 100 nm/min. Dotted vertical lines at 218 nm and 206 nm represent the characteristic wavelengths of β-sheet and α-helix.

Figure S2. Tryptophan fluorescence emission spectra of wild type and F10_Y11delinsLN mutant human γS-crystallin in increasing concentrations of GuHCl. A) Wild type γS-crystallin. B) F10_Y11delinsLN mutant γS-crystallin. λ_{exc}: 295 nm. Dotted vertical lines represent emission maxima of the proteins under each level of GuHCl stress.

Figure S3. Further analysis of thermal denaturation curves. A) First derivatives of the thermal unfolding curves of wild type (black line) and F10_Y11delinsLN mutant (brown circles) γS- crystallin monitored by tryptophan emission and shown in Fig. 3C. B) Van't Hoff plot of wild type (black squares) and F10_Y11delinsLN mutant (brown circles) γS-crystallin unfolding curves generated from data shown in Fig. 3A for CD-unfolding and C) Van't Hoff plot calculated for the unfolding curves shown in Fig. 3C for tryptophan emission. Solid lines represent the two-state fit. D) Wild type γS-crystallin and E) F10_Y11delinsLN mutant γS-crystallin CD spectra under thermal stress. MT: mutant.

Figure S4. Association and spectroscopic properties of αA-crystallin. A) Analytical size exclusion chromatography profile of wild type αA-crystallin. Protein concentration: 75 µM. B) Far-UV CD spectrum of wild type αA-crystallin. Scanning speed: 100 nm/min. Dotted vertical lines at 218 nm and 206 nm represent the characteristic wavelengths of β-sheet and α-helices, respectively. C) Near-UV CD spectrum of wild type αA-crystallin. Protein concentration: 50 µM; Scanning speed: 100 nm/min. Dotted vertical lines represent tryptophan (295 nm), tyrosine (288 nm) and phenyl alanine (271, 265, 259 nm) CD maxima in the protein. D) Tryptophan fluorescence emission spectrum of wild type αA-crystallin. Protein concentration: 0.2 mg/mL; λ_{exc}: 295 nm; excitation emission slits: 2.5 nm. Dotted vertical lines represent emission maxima of the proteins.

Figure S5. Measurement of particle size distribution after thermal stress by dynamic light scattering intensity distribution. A) Wildtype γS-crystallin. B) Wild type γS-crystallin + αA-crystallin 1:1 molar ratio. C) F10_Y11delinsLN mutant γS-crystallin. D) F10_Y11delinsLN mutant γS-crystallin + αA-crystallin 1:1 molar ratio. E) αA-crystallin alone. wt: wild type γS-crystallin, MT: F10_Y11delinsLN mutant γS-crystallin, αA: αA-crystallin.

Table S1. ESI MS analysis of SEC column fractions. A) Detected mass details of room temperature wild type human γS-crystallin+αA-crystallin size exclusion chromatography fractions presented in figure 7A. B) Detected mass details of room temperature mutant human γS-crystallin F10_Y11delinsLN+αA-crystallin size exclusion chromatography fractions presented in figure 7B. C) Detected mass details of 50° C wild type human γS-crystallin+αA-crystallin size exclusion chromatography fractions presented in figure 7A. D) Detected mass details of 50° C mutant human γS-crystallin F10_Y11delinsLN+αA-crystallin size exclusion chromatography fractions presented in figure 7B. E) Detected mass details of 60° C wild type human γS-crystallin+αA-crystallin size exclusion chromatography fractions presented in figure 7A. F) Detected mass details of 60° C mutant human γS-crystallin F10_Y11delinsLN+αA-crystallin size exclusion chromatography fractions presented in figure 7B.
