# Supplementary figures and images for: Human γS-Crystallin Mutation F10_Y11delinsLN in the First Greek Key Pair Destabilizes and Impairs Tight Packing Causing Cortical Lamellar Cataract
Source: Int J Mol Sci. 2023 Sep 20;24(18):14332. doi: 10.3390/ijms241814332 (PMC10531703; doi:10.3390/ijms241814332)

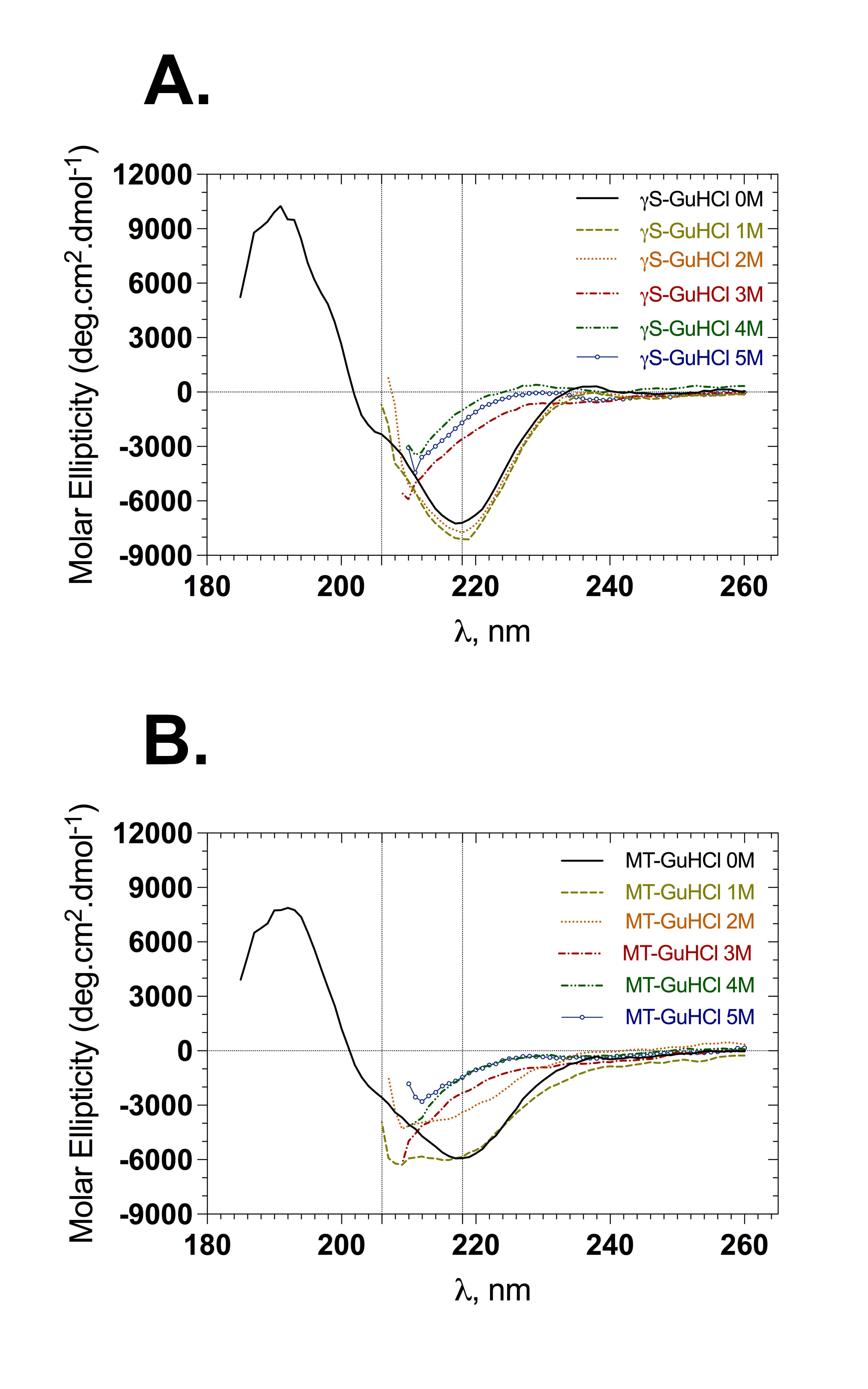

Supplement: Supplementary file 1 [file ijms-24-14332-s001.zip › Figure S1 copy.jpg]

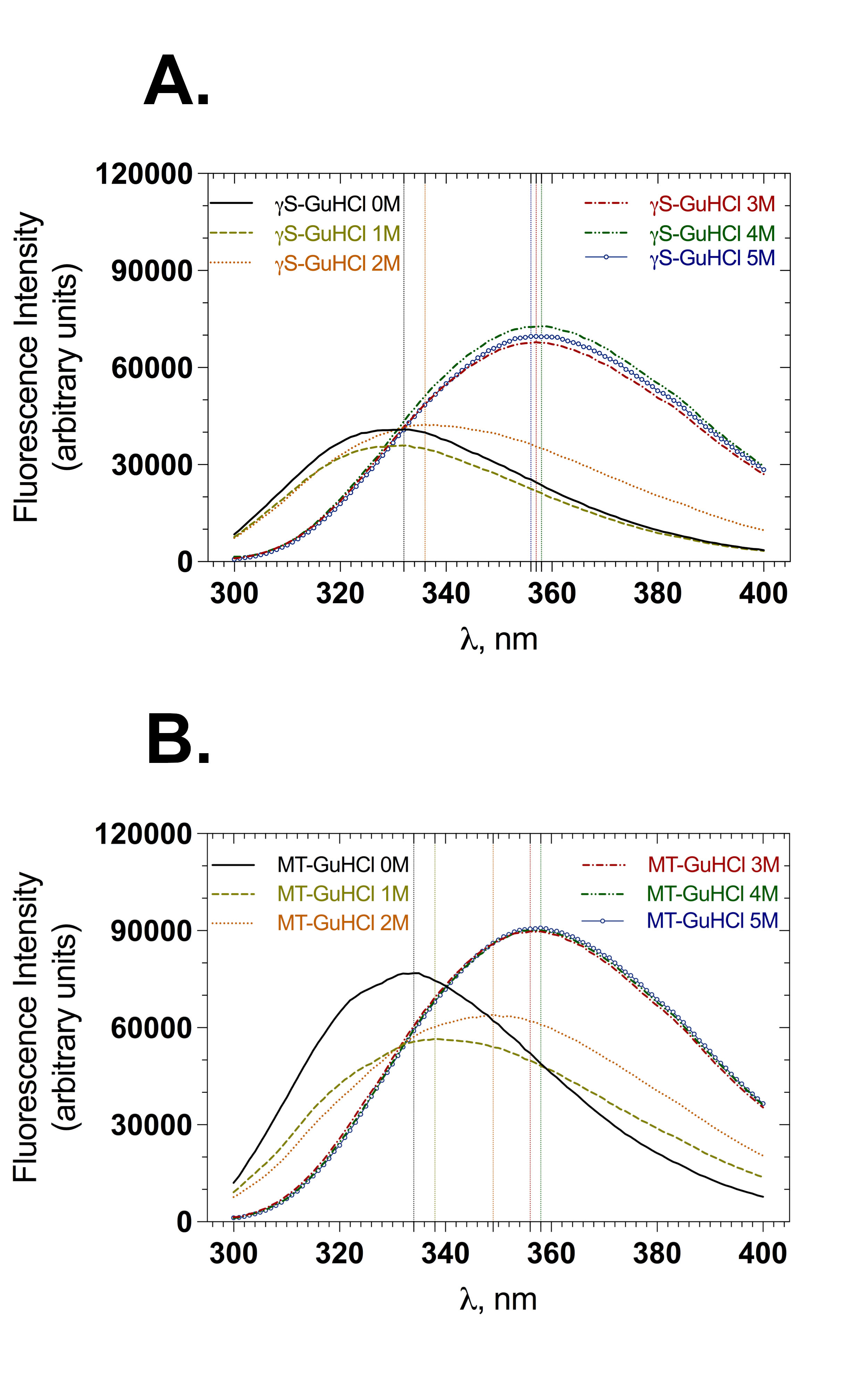

Supplement: Supplementary file 1 [file ijms-24-14332-s001.zip › Figure S2 copy.jpg]

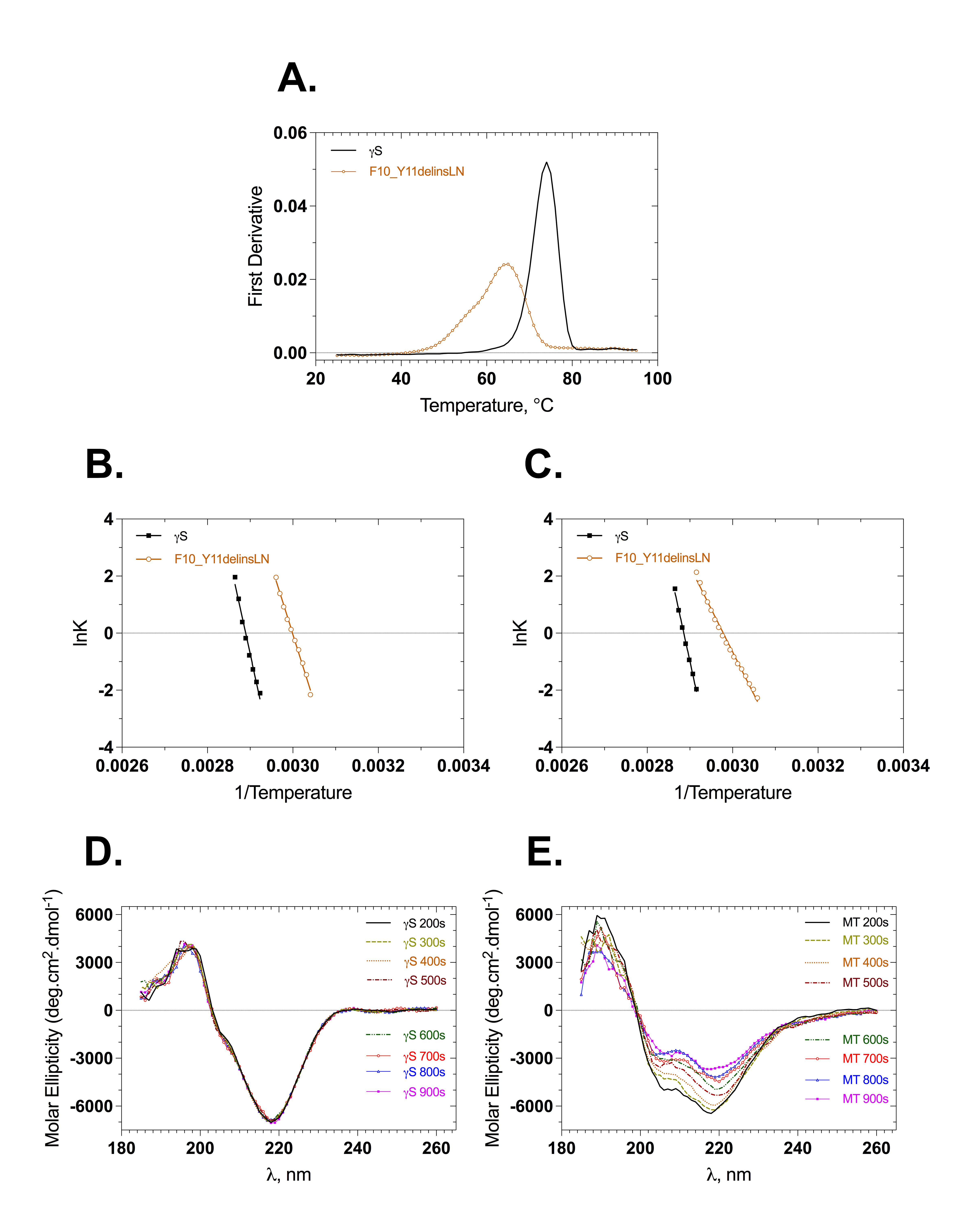

Supplement: Supplementary file 1 [file ijms-24-14332-s001.zip › Figure S3 copy.jpg]

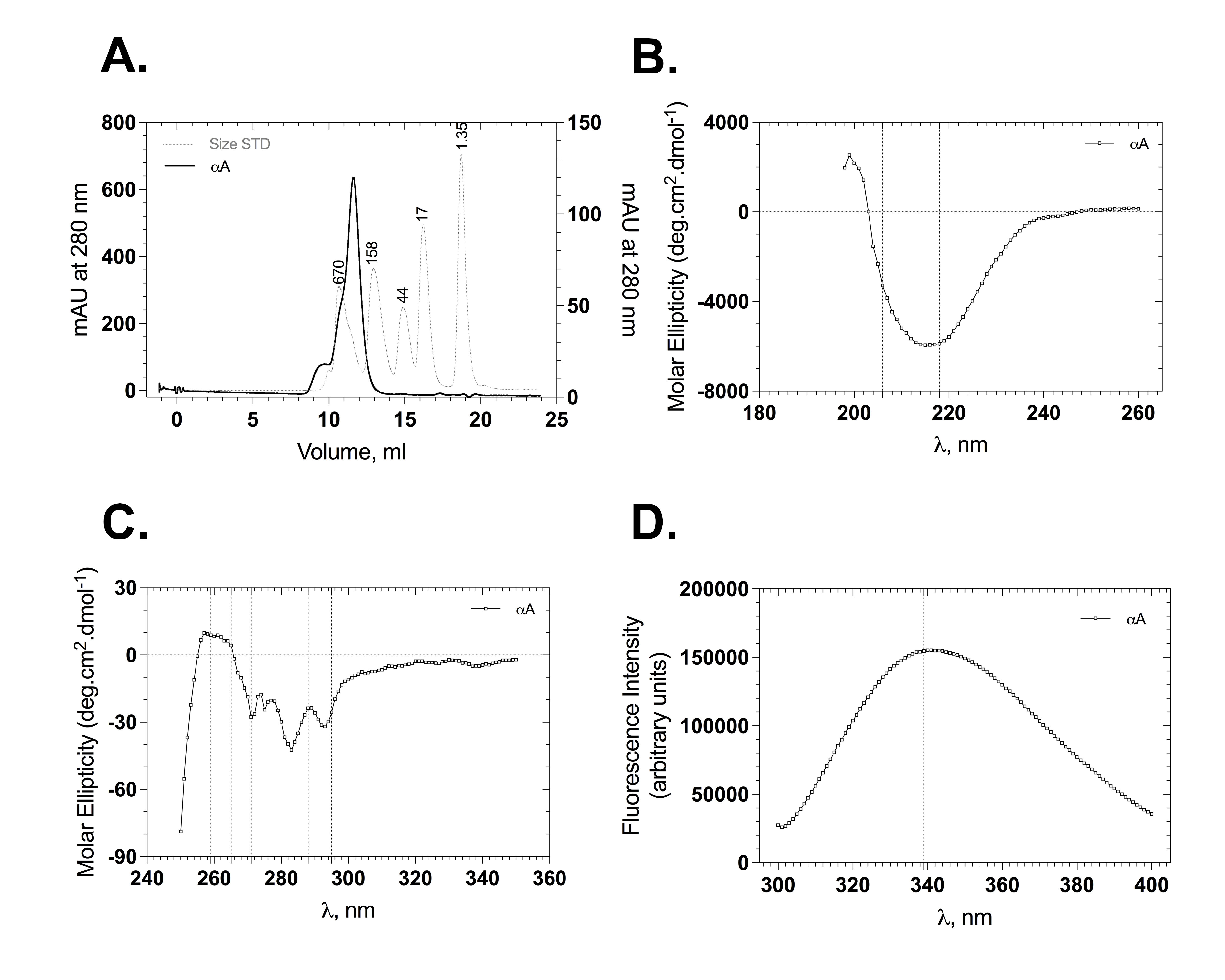

Supplement: Supplementary file 1 [file ijms-24-14332-s001.zip › Figure S4 copy.jpg]

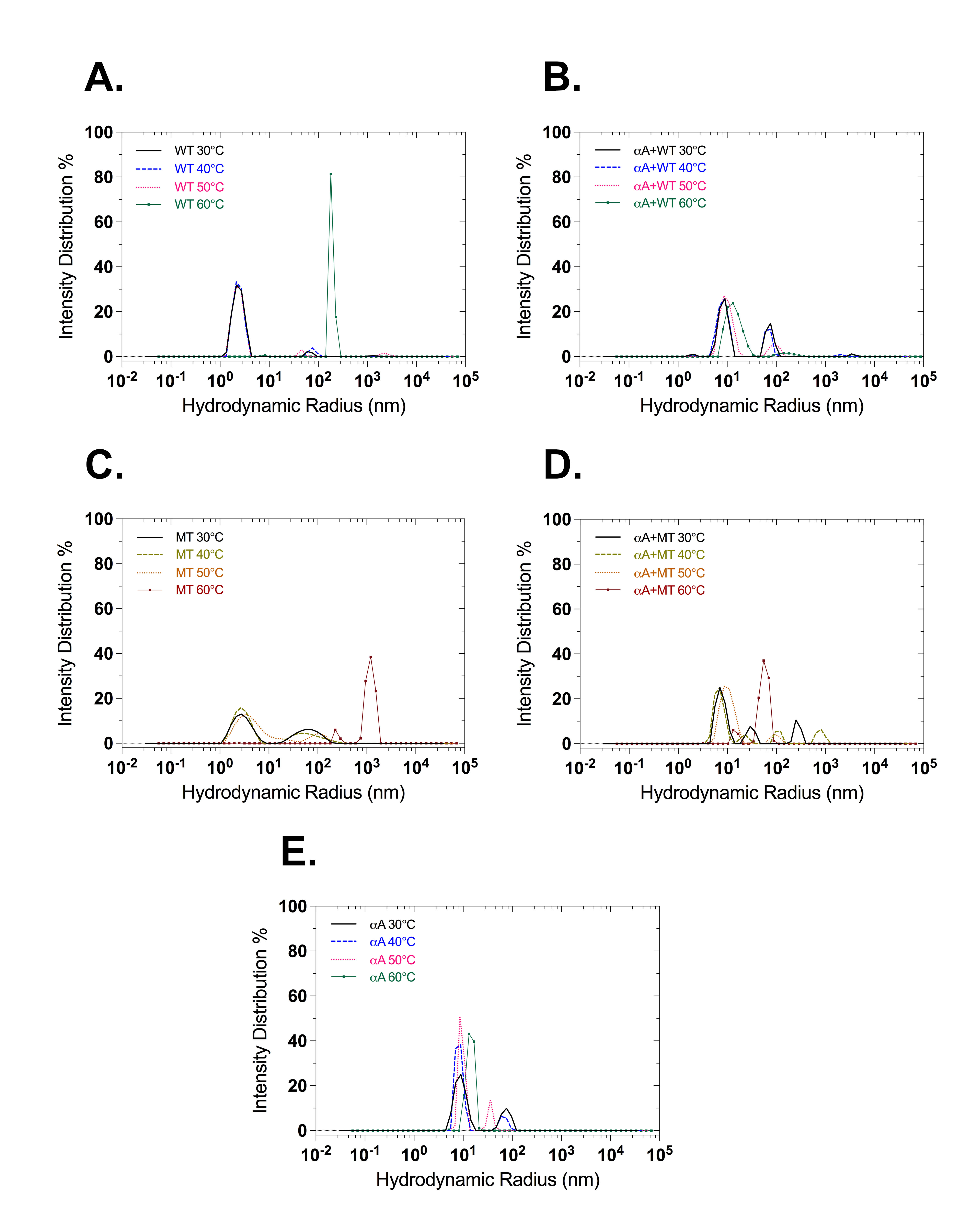

Supplement: Supplementary file 1 [file ijms-24-14332-s001.zip › Figure S5 copy.jpg]
